# Fitbit Data to Assess Functional Capacity in Patients Before Elective Surgery: Pilot Prospective Observational Study

**DOI:** 10.2196/42815

**Published:** 2023-04-13

**Authors:** Alessandra Angelucci, Massimiliano Greco, Stefano Canali, Giovanni Marelli, Gaia Avidano, Giulia Goretti, Maurizio Cecconi, Andrea Aliverti

**Affiliations:** 1 Dipartimento di Elettronica Informazione e Bioingegneria Politecnico di Milano Milano Italy; 2 Department of Biomedical Sciences Humanitas University Pieve Emanuele Italy; 3 Department of Anesthesiology and Intensive Care IRCCS Humanitas Research Hospital Rozzano Italy; 4 META - Social Sciences and Humanities for Science and Technology Politecnico di Milano Milano Italy

**Keywords:** wearable devices, smartwatch data, preoperative risk assessment, ethics in wearables, mobile phone

## Abstract

**Background:**

Preoperative assessment is crucial to prevent the risk of complications of surgical operations and is usually focused on functional capacity. The increasing availability of wearable devices (smartwatches, trackers, rings, etc) can provide less intrusive assessment methods, reduce costs, and improve accuracy.

**Objective:**

The aim of this study was to present and evaluate the possibility of using commercial smartwatch data, such as those retrieved from the Fitbit Inspire 2 device, to assess functional capacity before elective surgery and correlate such data with the current gold standard measure, the 6-Minute Walk Test (6MWT) distance.

**Methods:**

During the hospital visit, patients were evaluated in terms of functional capacity using the 6MWT. Patients were asked to wear the Fitbit Inspire 2 for 7 days (with flexibility of –2 to +2 days) after the hospital visit, before their surgical operation. Resting heart rate and daily steps data were retrieved directly from the smartwatch. Feature engineering techniques allowed the extraction of heart rate over steps (HROS) and a modified version of Non-Exercise Testing Cardiorespiratory Fitness. All measures were correlated with 6MWT.

**Results:**

In total, 31 patients were enrolled in the study (n=22, 71% men; n=9, 29% women; mean age 76.06, SD 4.75 years). Data were collected between June 2021 and May 2022. The parameter that correlated best with the 6MWT was the Non-Exercise Testing Cardiorespiratory Fitness index (*r*=0.68; *P*<.001). The average resting heart rate over the whole acquisition period for each participant had *r*=−0.39 (*P*=.03), even if some patients did not wear the device at night. The correlation of the 6MWT distance with the HROS evaluated at 1% quantile was significant, with Pearson coefficient of −0.39 (*P*=.04). Fitbit step count had a fair correlation of 0.59 with 6MWT (*P*<.001).

**Conclusions:**

Our study is a promising starting point for the adoption of wearable technology in the evaluation of functional capacity of patients, which was strongly correlated with the gold standard. The study also identified limitations in the availability of metrics, variability of devices, accuracy and quality of data, and accessibility as crucial areas of focus for future studies.

## Introduction

### Background

Every year, in the world, >300 million surgical procedures are performed, both for inpatients and outpatients [1,2]. Surgical operations are subject to the risk of complications owing to various concurrent phenomena and are aggravated by the patient’s preexistent health status. For elective surgeries, which aim at solving a nonimmediate life-threatening condition and are planned, numerous methods of patient evaluation in the preoperative phase are adopted to estimate cardiorespiratory function, hence the risk of complications to allocate the limited resources of the hospital in terms of staff, equipment, and time in the appropriate manner. Functional capacity is specifically important for patients who are old and frail, who are at increased risk for postoperative complications [3]. The information provided by exercise testing is often used to inform patients’ and providers’ decisions and target appropriate perioperative management [4].

In clinical practice, the focus of the preoperative assessment is on functional capacity, and traditional tests and measures are about physical activity and related metrics. They include objective tests, such as the 6-Minute Walk Test (6MWT) [5] and the cardiopulmonary exercise testing [6], and evaluation scales that rely on the subjective judgment of the physician or the self-evaluation of the patient. The 6MWT evaluates exercise tolerance in patients, and it is usually performed by any patient undergoing major surgery. It consists of making the patient walk at a self-paced speed up and down a 30-m long corridor for a fixed period of 6 minutes. The patient should walk alone, and no incitement should be done—only standardized phrases should be used to speak to the patient [5]. As a primary result of the test, the total distance traveled in meters (6MWT distance; 6MWD) is indicated. A normal value for healthy adults is 400 to 700 m [7], whereas exercise tolerance is considered to be reduced when <350 m is covered, even though there is no unanimity about result interpretation, and a difference of 50 m is considered to be substantial [8]. Secondary measures are heart rate (HR), respiratory rate, and arterial blood oxygen saturation, monitored each minute of the test and 2 minutes after the recovery phase has started. Short distance, high HR, and low oxygen saturation are fairly correlated with morbidity and mortality, even though low 6MWD is nonspecific and nondiagnostic because it does not provide information regarding the mechanism of exercise limitation [7]. The advantages of the test are simplicity, absence of need for sophisticated equipment, broad applicability, tolerability, and comparability with daily activity. A relevant limitation is the high variability, both between and within individuals, of the result owing to several factors. The motivation of the patient and other uncontrollable psychological factors may undermine the reproducibility of the test because it is self-paced [9].

Evaluation scales include the Metabolic Equivalent of Task [10], Clinical Frailty Scale [11], and Duke Activity Status Index [12]. The Metabolic Equivalent of Task is a simple 10-point scale on which patients rate the maximum level of activity they are able to perform, from sitting to vigorous activity [10]. The Clinical Frailty Scale [13] and Duke Activity Status Index [12] are two standardized clinical scales for preoperative risk assessment. Preoperative anesthesiologic examinations include the American Society of Anesthesiology Physical Status scale, the most widely used risk scale in anesthesia. Another index used to evaluate the fitness level of a patient is the Non-Exercise Testing Cardiorespiratory Fitness (NET-F), developed by Stamatakis et al [14] as a combination of measures, obtainable with all information usually available from the electronic health record and the estimate of the patient’s physical activity. This value is a weighted sum of coefficients derived from sex, age, BMI, resting HR (RHR), and physical activity level (PAL). NET-F was proven to be a robust predictor of mortality, achieving better performances than its individual components. Objective tests are highly time consuming, whereas the listed scales are partly or completely subjective.

In clinical settings, other parameters that allow the evaluation of the patient belong to the domains of physical activity, heart health, respiratory health, and sleep, among others. Physical activity guidelines recommend a moderate level of exercise at all ages and define threshold values for steps walked and energy expenditure [15]. Cardiac pathologies are the leading cause of death in developed countries, and measures of HR and HR variability (HRV) are primary indicators of such pathologies [16]. Sleep is an important natural state, and it influences most systems of the body. Respiratory rate has been found to be more accurate than pulse rate and blood pressure in predicting unfavorable clinical events and distinguishing between patients who are stable and those who are at risk [17].

### Wearables—Benefits and Current Limitations

A breakthrough innovation in the field of objectively assessed patient’s overall health, and in particular, preoperative functional capacity, may come from wearable devices, which find many applications in the fields of telemonitoring of patients [18], consumer health and well-being, sports performance assessment [19], environmental monitoring [20], and emergency settings [21]. Data collection refers to a large set of diverse clinical domains, and the approach of measurement of the devices for a selected parameter can vary substantially. Wearables exist for different parts of the body, allowing the estimation of different quantities. Examples of wearables include watches (such as smartwatches and activity trackers), eyeglasses, visors [22], rings [23], patches [24], sensorized garments [25], elastic bands applied on the torso [26], and earbuds.

Physiological measurements can consider a single parameter, for example, HR, peripheral blood oxygen saturation, respiratory rate [27], and skin temperature, or could be more complex measures combining data from several sensors, such as in the cases of sleep analysis, estimation of energy expenditure, and analysis of physiological parameters with respect to the activity [28]. Even pathophysiological conditions could be accurately detected after appropriate processing of the data, for instance, atrial fibrillation detection [29], high serum glucose level detection, and fall detection [30].

Innovative indexes that can be obtained using wearables have been defined in the literature. For instance, a Fitbit device was used by Mishra et al [31] to detect the infection by SARS-CoV-2 before the appearance of symptoms. To do so, the HR over steps (HROS) index was computed to detect anomalies in the heart’s behavior in relation to the level of activity.

In recent years, the entry of global companies such as Google, Apple, Samsung, Sony, Philips, and others into the field of wearable technology, particularly smartwatches, has accelerated the development of low-cost, attractive, and reliable devices. These types of smartwatches can measure many physiological parameters and can connect with the smartphone and internet, thus creating a so-called body area network [17] for a large number of users.

Along with their promises and benefits, wearables present some critical aspects that have been arising with the development and diffusion of this recent technology [32].

The number of wearable devices entering the market has been growing, but most of these are commercial devices, not research or medical devices, and not all of them are capable of measuring health parameters in a reliable manner.

Owing to design simplicity or manufacturing flaws, the devices able to grant a medical-grade measure are only a subgroup of the ones available. The quality of the data created by these devices should be proven to be sufficiently high to be helpful in the decision-making process and to draw medical conclusions. This is especially true for data-intensive methods, benefits of which strongly rely on the transparency, validity, and quality of data practices.

In addition, the competition in consumer technology might lead to the development of proprietary technological solutions and classification algorithms to convert the raw signals from the sensors into biomedical data. Although simple algorithms, for instance, to detect steps using a 3-axis accelerometer, are adopted consistently and similarly in most devices, more complex features, such as physical activity recognition and sleep stage classification, are often implemented substantially differently. This can make it difficult to verify whether the same measurement made by devices of two different brands refer to the same physical phenomenon and can lead to a lack of interoperability.

### Aim of the Study

As presented previously, preoperative assessment methods traditionally used in hospitals are burdensome and require many resources in terms of equipment or personnel, and subjective evaluation may compromise the accuracy of risk stratification.

The development and mass spreading of wearable activity trackers that allow the recording of physiological parameters in the domains of HR and energy expenditure may lead to a new low-risk strategy to objectively screen the population and test cardiorespiratory function before a surgical operation. This might permit the substitution of clinical practice involving qualified physicians in the preoperative assessment with an automatic data collection method without implications and need for supervision.

The aim of this study was to prove that useful health and clinical knowledge can be derived from low-cost commercial wearable device data. To do so, data from Fitbit Inspire 2 were collected and processed to create features that can highlight a stratification and correlate with the risk of complications, as traditional tests allow.

## Methods

### Device Characteristics

The Fitbit Inspire 2 [33] is a fitness tracker that was first released on September 25, 2020, as the new version of the low-cost entry-level series, Inspire. Its price was <US $100 at the time of the purchase and was chosen owing to several favorable characteristics: it is small and lightweight, thus can be worn without fatigue by older patients; its small display leads to prolonged battery life (no need to train the patient to recharge the device); and it is affordable and thus interesting for the health care system in both high-resource settings and low-resource settings.

Using the Fitbit app, available for Android and iPhone Operating System, users can enter their information about age, sex, height, and weight. The battery is a rechargeable lithium polymer, and the manufacturer declares life of up to 10 days, with a charging time of 2 hours from 0% to 100%.

Fitbit Inspire 2 is equipped with a 3-axis accelerometer for movement detection and an optical HR monitor (photoplethysmography [PPG] sensor) composed of green and infrared light emitting diodes and photodiodes.

The memory of the device allows storage of 7 days of detailed data (minute by minute) and daily totals for the past 30 days.

Users were not allowed to synchronize data with their personal mobile phones; all data were synchronized by the investigators when devices were retrieved. This solution was chosen because the patients participating in the study were old and generally had limited digital literacy. In addition, installing an app by a commercial third party on the smartphone of patients would have caused problems in terms of data protection.

As the users could not synchronize the devices with the mobile phone, some functionalities such as GPS tracking were not available during the tests. Furthermore, the lack of continuous synchronization results in data loss in case the acquisition lasted longer than 7 days; however, the flexibility of –2 to +2 days was needed for organizational purposes.

After synchronizing the device with the mobile app, data were uploaded to the cloud repository and were accessible to download through application programming interfaces on the web panel. The data that are used in this study are those related to the HR and activity, and sleep data are not used in this context. Few studies reported that sleep duration estimated by Fitbit devices is a reliable indicator, whereas classification of sleep stages has insufficient accuracy [31,34].

### Experimental Protocol

Patients were selected according to the surgical intervention they were scheduled for, taking into consideration major noncardiac surgeries with general anesthesia (eg, spinal surgery, mastectomy, and gallbladder resection). Enrollment of patients started in June 2021 and ended in May 2022. Other inclusion criteria were age >70 years and no mobility deficit or orthopedic problems to safely perform the walk test.

Routinely registered information about the patient was collected: age, sex, weight, height, clinical information about comorbidities, type and characteristics of the surgical intervention, and laboratory analyses result if required.

Patient’s functional status was evaluated using the 6MWT during a visit before the operation.

After the visit, if patients accepted to participate in the study, they were given the wearable activity tracker Fitbit Inspire 2. Patients were asked to wear the Fitbit Inspire 2 for 7 days (with flexibility of –2 to +2 days) after the hospital visit, before their surgical operation.

The physician or the medical student who enrolled the patient had to update the user’s general information, that is, age, sex, weight, and height, through the Fitbit app, to calibrate the internal algorithms for proper data collection.

### Data Preprocessing

Of the 31 enrolled patients, 2 (6%) patients did not wear the device at night; therefore, HR and sleep data are not available. Of the 31 patients, 2 (6%) patients interrupted the recording on the fourth day and 1 (3%) delayed the beginning by 3 days. A patient wore a smartwatch that by mistake was set to be *on-clip*; thus, the PPG system was switched off—no data about HR and sleep were recorded. For this patient, a reduced processing pipeline was created, considering only data about step count and substituting the Fitbit-evaluated RHR with the HR recorded at the hospital visit before the 6MWT. A patient wore the device with wrong characteristics set through the app; therefore, activity data might be mistaken; nevertheless, these data were aggregated with the others.

The actual duration of the experiment was on average 7.03 (SD 2.3) days, very similar to the expected periods, and only 6% (2/31) of the patients wore the tracker for 3 days, and 3% (1/31) wore it for 14 days.

### Data Processing and Feature Engineering

Some parameters are directly computed by the Fitbit, such as HR and steps, whereas other indexes can be derived or estimated from the downloadable data. Data about the HR, physical activity, and sleep were transformed and combined to allow patient risk stratification in anticipation of the elective surgical operation.

#### HR and Health Metrics

HR is continuously estimated through the PPG sensor, the raw data of which are processed by an internal proprietary algorithm. Output HR data are presented at 1-second intervals during physical exercise tracking and at 5-second intervals at all other times. HR measurement is affected by the location of the device and movements. RHR is computed on a 24-hour basis. It is known from the literature that this value reaches its minimum at night [35]. Therefore, nighttime values of RHR are comparable between patients, whereas daytime values might not reach the minimum. In the case of patients who did not wear the device at night and had no RHR data available, the values were substituted with the HR measured by the clinician before the 6MWT.

HR zones are presented as customizable ranges that are set subdividing the HR reserve, that is, the difference between maximal HR and RHR. During a workout, entering a different HR zone is announced through a vibration. HRV quantifies the extent to which the respiratory rate interval or HR changes from one cardiac cycle to the next. This can provide us a lot of information about how the autonomic nervous system regulates the heart. HRV has been correlated with cardiac diseases [36], fitness, and functional reserve [37] and is positively affected by exercise [38]. During our study, no HRV values were made available for download, even if the user manual of the device mentions it among the advanced health metrics provided by the device [33]. HRV could not be derived from the raw data also, because no R-R peak distance information is given.

#### Activity

Number of walked steps and distance covered are computed using the 3-axis accelerometer only, as processing algorithms can estimate the cadence. A traditional threshold of 10,000 daily steps is well accepted as indicative of an active lifestyle [39], and it is the threshold used in the *Results* section of this paper. Distance is computed as the number of steps multiplied by the stride length (based on sex and height information entered by the user).

The time spent in physical activity is grouped in 4 categories based on intensity and reported as a sum: *sedentary*, *light*, *moderate*, and *very active*. Active zone minutes are a point-based metric that counts the time spent in different activities, classified and listed in order as *fat burn*, *cardio*, and *peak*. An overall cardio fitness score, an approximation of cardiovascular fitness as maximal oxygen consumption, is also estimated by considering the user’s age, sex, BMI, and RHR.

#### HROS Feature

HROS is a feature that can be computed simply as the instantaneous ratio between HR and steps. HR is generally reported every 5 seconds, whereas the value of steps is sampled every minute by the device. This value is increased by 1 with the aim to avoid the division by 0 during sedentary situations. The value of steps is considered to be constant in a minute, whereas the available HR data are used. This gives an estimate of the effort and functional capacity of the patient, as high value of HROS indicates that the heartbeat is fast while the intensity of the physical activity is moderate, and low value indicates a restrained heartbeat while facing challenging physical activity.

This feature was used in a previously mentioned study for early detection of SARS-CoV-2 infection before the appearance of symptoms [31]. The authors of the study developed a structured algorithm for computing HROS and detecting anomalous values, through a dispersion approach that considers the deviation from a normal distribution by means of an elliptic envelope method. The code to process data was retrieved by the GitHub repository associated with the study by Mishra et al [31]. The anomalies are detected using a contamination, that is, the portion of outliers in the data set, of 0.1. In this manner, the algorithm identifies some outliers in the computed feature, corresponding to approximately 10% of the total. This value was chosen based on the method described in the original paper [31], that is, an iterative approach with increasing contamination values until a consistent number of outliers is detected.

In our study, HROS was evaluated at 1% quantile. The 1% quantile was chosen to account for the possibility that the minimum HROS was an outlier.

#### NET-F Feature

In the previously cited article by Stamatakis et al [14], a measure named NET-F was developed as an estimate of maximal oxygen consumption, which does not require performing an actual physical test. It is an indicator computed as a weighted sum of information routinely collected and available from the electronic health record, that is, sex, age, BMI, measured RHR, and self-reported PAL obtained by questioning the patient.

The formula to compute the metric is the following:


NET – F = sex × 2.87 – (age × 0.11) – (BMI × 0.17) – (RHR × 0.05) + (PAL) + 21.41

Where sex takes value of 1 for men and 0 for women, age is expressed in years, BMI is computed as weight (kg) over the square of height (cm), RHR is expressed in beats per minute and measured during a hospital visit, and PAL is based on the weekly frequency of moderate to vigorous physical activity (MVPA) sessions—1 session is defined as either 30 minutes of moderate-intensity activity or 15 minutes of vigorous-intensity activity. The PAL value has 5 different levels:

1. Level 1—no physical activity; coefficient=0
2. Level 2—<1.5 MVPA sessions per week; coefficient=0.35
3. Level 3—1.5 to <3 MVPA sessions per week; coefficient=0.29
4. Level 4—3 to 6 MVPA sessions per week; coefficient=0.64
5. Level 5—>6 MVPA sessions per week; coefficient=1.21

The frequency of weekly physical activity was obtained through a series of questions to the patient. The latter is the most heavily weighted variable, and it was assessed using subjective self-evaluation. The authors state that “an NET-F method containing an objectively assessed physical activity component would have been an even more powerful feature.”

In our study, we used data obtained from the Fitbit instead of clinical data. RHR values are estimated daily and can be downloaded directly from the Fitbit device. With a wearable, it is possible to measure the physical activity performed by the patient during the week under study—the Fitbit records daily minutes of physical activity in the different active zones. By counting the number of sessions of 30 minutes of *moderately active minutes* and sessions of 15 minutes of *very active minutes*, PAL of each patient can be evaluated.

### Statistical Analysis

The analysis of the relationships between descriptive variables and outcomes is presented in two manners. First, the Pearson correlation between the primary outcome and the Fitbit feature is computed.

Then, a threshold was searched for HROS evaluated at 1% quantile and NET-F to discriminate between a *good* and a *bad* 6MWD (≥350 m and <350 m, respectively), with the aim of maximizing the difference in the primary outcome between them. To test the difference in the distribution of a variable in the 2 populations, the Wilcoxon rank sum test was used. To find an optimal threshold that can distinguish between the 2 groups, the receiver operating characteristic (ROC) curve was exploited, by plotting the results of true positive rate (TPR) and false positive rate (FPR) and choosing the value representing the best trade-off between a high TPR and a low FPR.

### Clinical Outcomes

This study aimed at finding a method to manage data obtained from wearable device, Fitbit Inspire 2, and create useful knowledge for patient risk stratification. To do so, the reference gold standard measure for patient assessment was the 6MWD, measured in meters and collected by the medical staff during the same visit during which the Fitbit is given to the patient. This was the primary clinical outcome of the study. In this study, 350 m was considered as the threshold of 6MWD.

### Ethics Approval

The study was approved by the Humanitas Research Hospital Ethical Committee (institutional review board number 350/21; April 20, 2021) and was registered in clinicaltrials.gov (NCT05083598). Written informed consent was collected from all patients.

## Results

### Overview

This section presents the results of the study. Correlation with the primary outcome, the 6MWT walked distance, is reported. A comparative analysis is outlined, determining differences among populations defined by several criteria. Finally, inconsistencies in the data are discussed.

In total, 31 patients were enrolled in the study. Patients’ characteristics, clinical scales results, and physiological parameters measured during the 6MWT are summarized in Table 1.

**Table 1:** Patients’ characteristics, clinical scales results, and physiological parameters measured during the 6MWT^a^.

	Total (N=31), mean (SD)	Women (n=9, 29%), mean (SD)	Men (n=22, 71%), mean (SD)
Age (years)	76.06 (4.75)	75.11 (5.21)	76.45 (4.63)
Height (cm)	167.87 (11.07)	156.78 (10.23)	172.41 (7.81)
Weight (kg)	70.39 (12.47)	61.33 (11.81)	74.09 (10.93)
BMI (kg/m^2^)	24.83 (2.61)	24.73 (1.90)	24.87 (2.89)
6MWT (m)	328.94 (99.53)	279.33 (138.76)	349.23 (73.01)
Mean HR^b^ (bpm^c^)	80.20 (15.93)	84.79 (16.20)	78.32 (15.81)
Resting HR (bpm)	73.03 (13)	77.78 (11.80)	71.09 (13.22)
Maximum HR (bpm)	90.68 (20.31)	95.56 (21.48)	88.68 (19.99)
Mean SpO_2_^d^ (%)	95.24 (1.76)	94.85 (1.69)	95.40 (1.81)
Resting SpO_2_ (%)	97.16 (1.19)	96.78 (1.09)	97.32 (1.21)
Minimum SpO_2_ (%)	92.58 (2.94)	91.22 (3.87)	93.14 (2.36)
RR^e^ at start (bpm)	13.71 (1.78)	13.79 (1.99)	13.56 (1.33)
RR at end (bpm)	17.14 (3.05)	16.74 (2.84)	18 (3.46)

^a^6MWT: 6-Minute Walk Test.

^b^HR: heart rate.

^c^bpm: beats per minute (when referring to heart rate) or breaths per minute (when referring to respiratory rate).

^d^SpO_2_: peripheral blood oxygen saturation.

^e^RR: respiratory rate.

### Correlation Analysis

**Figure 1:**
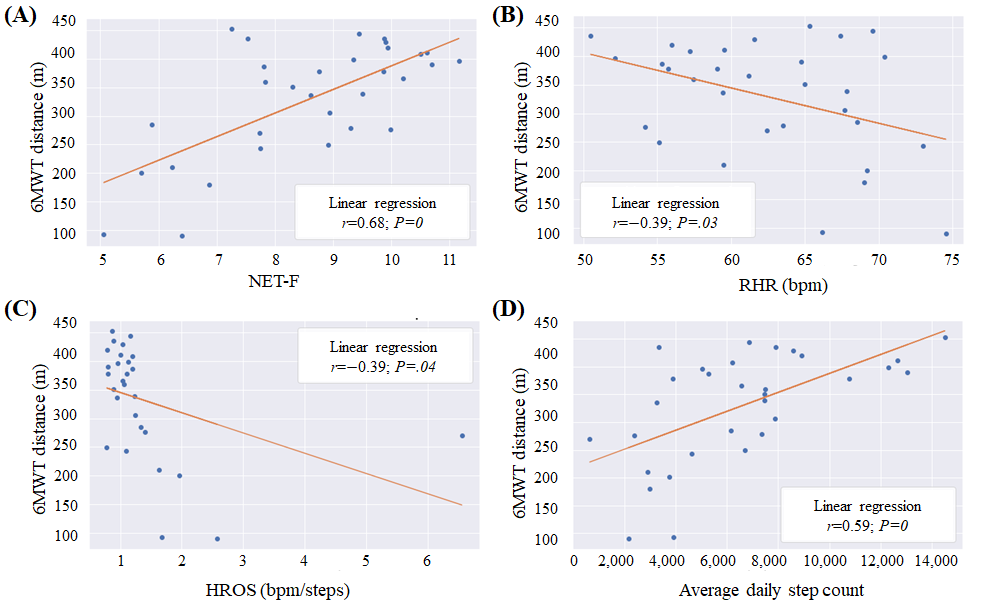
(A) Correlation between the 6-Minute Walk Test (6MWT) distance and Non-Exercise Testing Cardiorespiratory Fitness (NET-F), (B) correlation between the 6MWT distance and resting heart rate (RHR), (C) correlation between the 6MWT distance and heart rate over steps (HROS) at 1% quantile, and (D) correlation between the 6MWT distance and daily steps. bpm: beats per minute.

The parameter that correlated best with the 6MWT was the NET-F index (*r*=0.68; *P*<.001), computed as described in the *Methods* section and shown in Figure 1A.

Among the coefficients composing the NET-F feature, the wearable-derived PAL showed a moderate but significant correlation of 0.50 (*P*=.004), whereas the others had poor results. This is a confirmation of what the authors reported in a previously mentioned study [14]. Moreover, in the study by Stamatakis et al [14], the composed metric is more powerful than the self-reported PAL. However, in our study, the PAL is derived from wearable data; thus, it is an objective measure of the actually performed physical activity.

The RHR measured daily by the device proved to be a useful feature (Figure 1B). The average over the whole acquisition period for each patient had *r*=–0.39 (*P*=.03), even if some patients did not wear the device at night, which is the period during which the evaluation of this dimension is preferred.

The HROS feature expresses high fitness condition when its value is low. When the number of walked steps is very high, the HROS value can go below 1. The correlation of the 6MWD with the HROS evaluated at 1% quantile HROS was significant, with Pearson coefficient of −0.39 (*P*=.04; Figure 1C). In the figure, it can be noted that the linear correlation line slope would be steep, and the correlation would be high if the single outlier at the extreme right is removed.

Fitbit step count had a fair correlation of 0.59 with the 6MWT (*P*<.001) when considering the average number of daily steps (Figure 1D).

**Figure 2:**
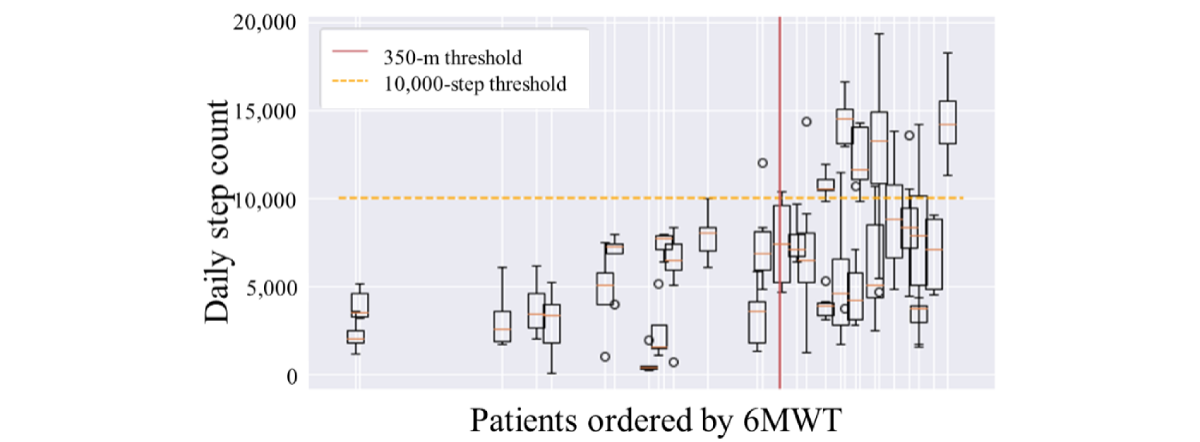
Daily step count with patients, ordered by 6-Minute Walk Test (6MWT) result.

Figure 2 shows the distribution of daily steps for each patient following the order of the 6MWT results on the x-axis. The horizontal threshold represents 10,000 daily steps, and the vertical red line indicates the 350-m threshold for the walk test. It is noticeable that patients below the 6MWT threshold have a consistently low number of steps, below the daily 10,000 steps, whereas patients on the right side of the graph have great variance and surpass the threshold more often.

To evaluate the performance of patients, it is possible to consider the mean daily steps or the maximum daily steps, that is, the best value of steps achieved during the test period. The latter is more representative of the ability to walk a certain distance. If the mean number of steps per patient is considered, very few patients are above the threshold. The conditional probability of having 6MWT result <350 m if the mean number of steps is <10,000 is 0.53. The conditional probability of having 6MWT result <350 m if the maximum number of steps is <10,000 is 0.75.

### Comparative Analysis

Although several normative studies on optimal daily steps and RHR values are already available in the literature, it is of interest to determine thresholds of HROS and NET-F that might help to discriminate between patients’ fitness levels. As stated in the *Methods* section, the outcome for the area under the curve (AUC) analysis is to determine whether the patient performed a *good* or *bad* 6MWT (distance ≥350 m or <350 m, respectively).

**Figure 3:**
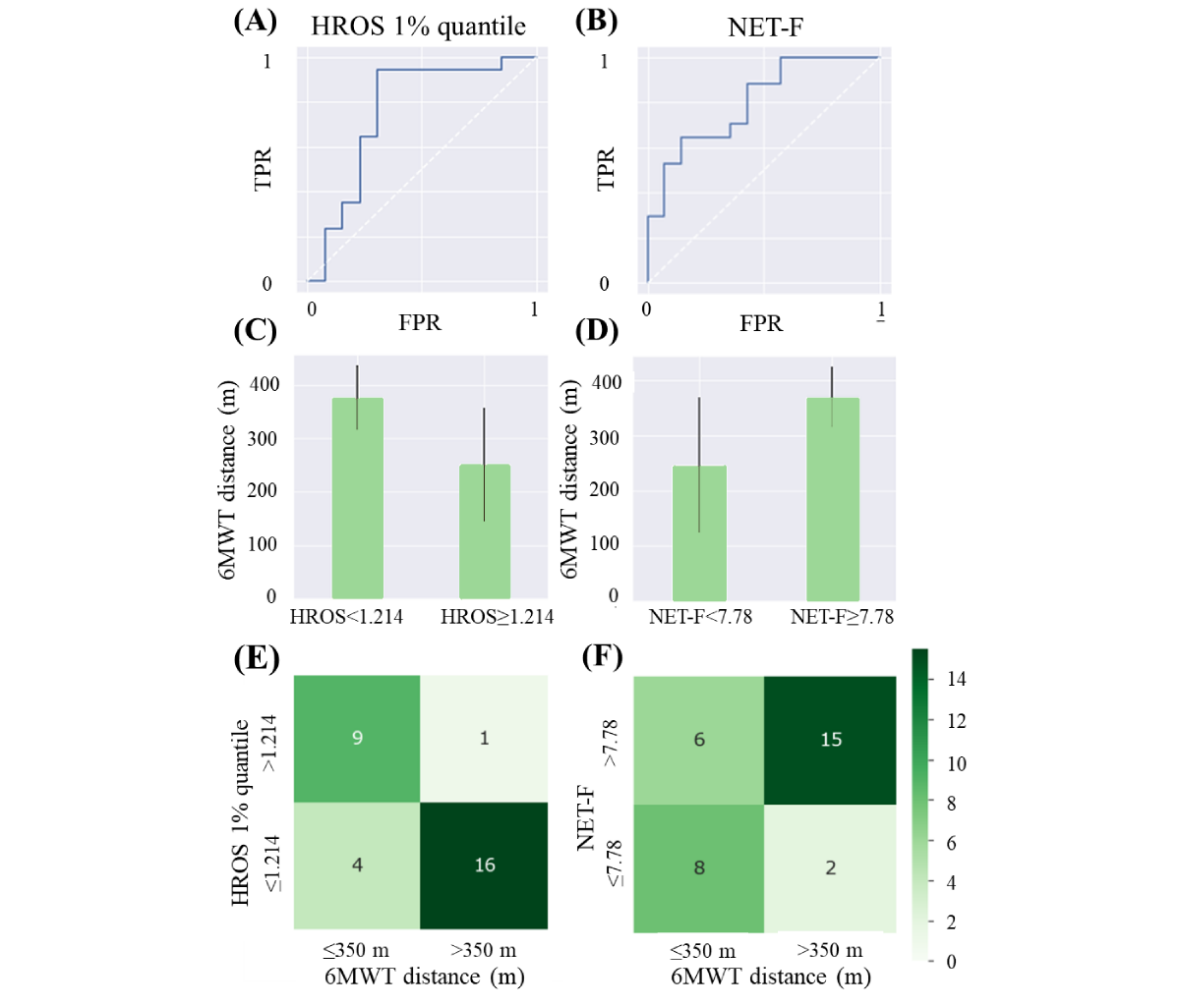
(A) Receiver operating characteristic (ROC) curve for 1% quantile heart rate over steps (HROS) threshold, (B) ROC curve for Non-Exercise Testing Cardiorespiratory Fitness (NET-F) threshold, (C) 6-Minute Walk Test (6MWT) distributions with chosen HROS threshold of 1.214, (D) 6MWT distributions with chosen NET-F threshold of 7.78, (E) contingency table of the 6MWT distance threshold of 350 m versus HROS 1% quantile threshold of 1.214, and (F) contingency table of the 6MWT distance threshold of 350 m versus NET-F threshold of 7.78. FPR: false positive rate; TPR: true positive rate.

Regarding the NET-F feature, the ROC curve has AUC of 0.80, and the optimal threshold, with TPR of 0.88 and FPR of 0.43, is 7.78. This value can significantly discriminate the 6MWT walked distance (*P*=.01). The ROC curve is shown in Figure 3B, and the 6MWT results for the 2 populations are shown in Figure 3D. The associated confusion matrix is shown in Figure 3F.

This threshold falls between the two thresholds proposed by Stamatakis et al [14]—9.8 and 6.8 for men and women, respectively, thus confirming the reliability of this feature.

A similar pipeline was adopted for the HROS feature, excluding 3% (1/31) of the patients owing to lack of data. Here, the approach involving 1% quantile had area under the ROC curve of 0.76, whereas the approach using the minimum value, besides including outliers in the evaluation, had poor AUC of 0.56. No threshold for this method was able to separate groups based on the 6MWD results. Instead, the version using 1% quantile highlighted a threshold of 1.24 beats per minute/step, which included 65% (20/31) of the patients in the fit group and classified 32% (10/31) of the patients as unfit (TPR=0.94; FPR=0.31). The ROC curve (Figure 3A) and the distributions of the primary outcome obtained using this method (Figure 3C) are represented. Moreover, in this case, the distributions are significantly different when tested with the Wilcoxon rank sum test (*P*<.001). The confusion matrix is shown in Figure 3E.

### Inconsistency of the Data

The data gathered in this study presented some inconsistencies among the populations and for certain measures. The reasons are different, attributable to patients’ compliance with the protocol, mistakes by the research staff, and Fitbit Inspire 2 itself. This led to the adaptation of some methods to analyze some data and the abandonment of some measures owing to implausibility.

Patients were instructed to wear the device for 7 days (with flexibility of –2 to +2 days); however, 6% (2/31) of the patients did not wear the device at night, probably because of discomfort reasons. This caused a deviation in the data format and required appropriate modifications to the code to process and analyze the data. Therefore, a different version of the pipeline was used for processing the data from this 6% (2/31) of the patients, for managing the lack of HR and RHR data during the night.

A similar process was adopted for a patient who wore a device with mistaken settings, causing the loss of HR recordings and all related measures, such as activity minutes.

**Figure 4:**
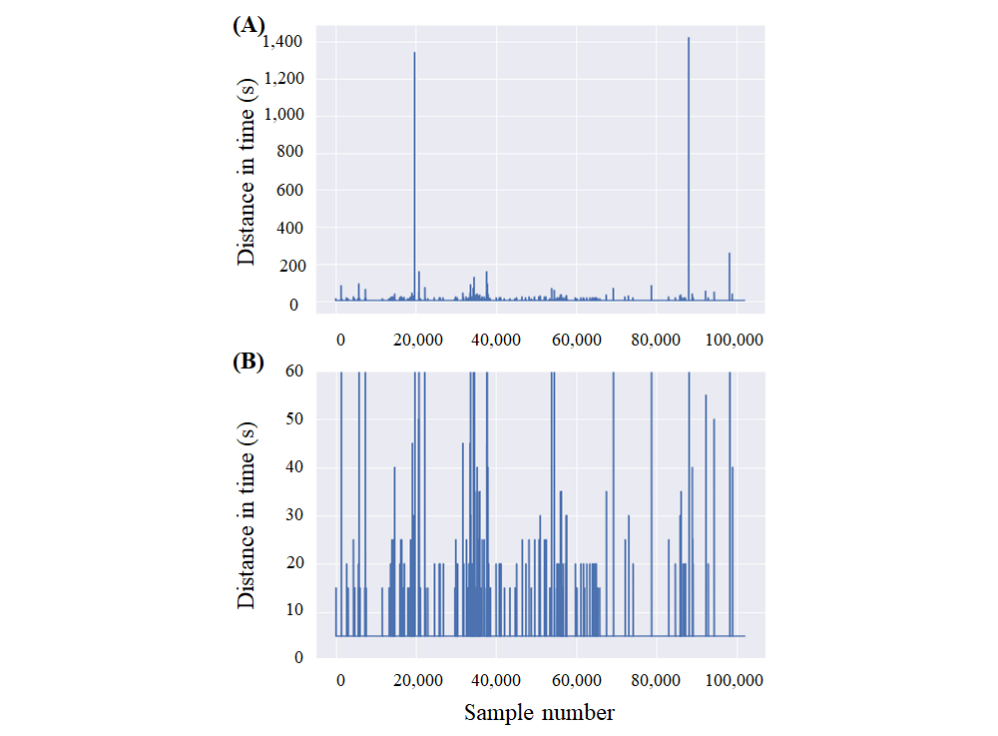
(A) Time distance of successive heart rate samples in a patient who was compliant with the protocol and (B) focus on the distribution of low distances.

HR data presented considerable gaps in the recording, even for continuous recordings. As stated by the manufacturer, the normal sampling period should be between 5 and 15 seconds; however, lack of data for >15 seconds was common. In Figure 4, the time difference between successive samples is shown for a patient who wore the smartwatch continuously. The image at the bottom is a zoomed-in version of the image on the top. Therefore, it was not possible to estimate HRV as no beat-by-beat information was available.

## Discussion

This section focuses on the achieved results of the study, then presents the main technological and ethical issues encountered in this specific study and in similar ones, and finally provides a short conclusion.

### Achieved Results

This study presents the limitations of observational research; it cannot establish causal relationships because no control group has been defined and information about the survival of the patients after the operation has not been gathered. However, it is useful to highlight the relationships between a widespread and well-accepted method of patient assessment (6MWT results) and measures obtained using a low-cost consumer-grade wearable device.

A strong correlation was found between the 6MWT and several measures obtained from the Fitbit. Simple measures, such as the RHR and daily step count, and composed indices, such as the NET-F and HROS, demonstrated close relationship with the primary outcome. Overall, the number of measures from this study that showed a relationship with gold standard methods used in preoperative assessment allows us to consider the wearable, Fitbit Inspire 2, as a promising starting point for the adoption of wearable technology in the evaluation of functional capacity of patients in clinical contexts.

Regarding the protocol of the experiment, the number of patients eligible for the study, also considering stringent criteria of age, total body anesthesia, and ability to perform the 6MWT, was limited.

Owing to their mobility problems or risky health conditions (eg, myocardial infarction), some patients could not be included in the study. This implies that patients at high risk generally do not perform the gold standard test at all in the clinical practice currently. However, this could be overcome by simply delivering a wearable device to such patients and asking them to wear it while performing normal daily life activities.

### Limitations and Future Developments

One of the main limitations of this study is the lack of HRV data, as this parameter is an indicator of cardiac health and fitness and provides prognostic information about clinical populations [40]. As explained in detail in the *Methods* section, HRV could not be computed owing to the lack of beat-to-beat time series, and no HRV data were available for download. In the Fitbit Inspire 2 user manual [33], it is written that this metric is available; however, on the website [41], there is a disclaimer that HRV is “only available in selected countries,” and this is a possible reason why we could not retrieve any data. The lack of HRV data appears to be easy to overcome, especially by using Fitbit smartwatches instead of activity trackers. It is possible to design custom apps for Fitbit smartwatches, but not for trackers, by exploiting the software development kit available. Research-grade Fitbits and particularly smartwatches also enable direct download of data from the device. These are major points in favor of using smartwatches instead of trackers for clinical studies. However, research-grade devices and smartwatches are more expensive than commercial trackers—this can be a problem, considering the potential for using wearable technology to cut costs and expand the availability of monitoring for the general population.

In addition, accuracy of the data obtained using the device, Fitbit Inspire 2, has not been extensively discussed in scientific literature. Most of the studies comparing the goodness of measure from a Fitbit wearable with a clinical-grade measure used different models, such as Charge, Surge, or Versa [42,43]. This variability raises concerns about interoperability also, as different wearable devices usually collect, label, and assess data in different ways, and it can be difficult to compare and verify the same measurement made by different devices.

The results of studies about the reliability of Fitbit smartwatches in clinical settings are inconsistent. Regarding HR measurement from a Fitbit wrist-worn device, a study [44] found that the accuracy of HR estimation from a PPG sensor (<10% mean absolute error with a gold standard electrocardiogram) across 24 hours and during various activities was acceptable. Other studies [45] instead reported a substantial variation in accuracy according to the type of activity performed, and a relevant percentage of HR measurements was substantially inaccurate. Regarding physical activity, a study from 2015 [46] found a strong correlation in step counts between measures of Fitbit and gold standard methods. However, they found low accuracy at slow walking pace, which is common in older adults, and pointed out the need for further studies involving the older population, which is the main population of this study. These results highlight the need to focus on data accuracy and quality as a central issue of wearable technology for health and the need to develop collaborative and common standards to define a framework for the assessment and promotion of data quality.

Accessibility was also one of the major issues in this study. The study was conducted independently from Google and Fitbit, and the chosen device is an activity tracker with no access to the firmware from outside. It was not possible to obtain the raw data from the accelerometer and the PPG, and only the structured information as output from internal proprietary algorithms was accessible. Although the download process from the Fitbit portal did not cause any problem, neither the design nor the correctness of the algorithms could be assessed. Accessibility is a crucial issue when assessing the accuracy, quality, and validity of wearables as a promising technology for health practitioners and biomedical researchers. However, accessibility is also crucial to assess the security and privacy of the health data collected through wearable technology and thus to determine the fairness and equity of wearable technology for patients.

All studies using wearable devices as activity trackers are limited in patients with mobility issues, such as those with lower limb fractures, those with peripheral vascular disease leading to limited ability to walk (ie, intermittent claudication), or patients who are bedridden. However, this limitation is shared by all methods of cardiovascular fitness assessment, including cardiopulmonary exercise testing and 6MWT. In these patients, the only option available is drug-induced stress testing, such as dobutamine stress echocardiogram, where cardiac reserve and possible inducible myocardial ischemia are tested through a powerful but reversible cardiac inotrope [47]. In this small proportion of patients who are at high risk, other types of wearable devices or prolonged monitoring over weeks should be proposed in the future.

### Conclusions

This study is the result of an observational study, and it was useful to highlight the relationships between widespread methods of preoperative assessment and data obtained using a low-cost commercial wearable device. This is a promising starting point for the adoption of wearable technology for the evaluation of patients in clinical contexts.

Future developments of the study should include a large pool of participants and assess the validity of other types of devices. Interoperability should be pursued for manufacturing techniques and processing algorithms, to allow the comparison of devices from different brands. An overarching and evidence-based consensus on the validity of these technologies is essential to integrate it in health care systems. A reflection on the epistemic role of wearables is due, with focus on the relative aspects of data quality, which is strictly bound to the context of application. Ethical implications should be analyzed, and regulations should be attentive to preserve patients’ interests.

There is a diffuse consensus that if developers, researchers, health care professionals, and regulatory institutions cooperate with the aim of finding solutions that focus on the needs and well-being of the patients, unique opportunities will be exploited, and breakthrough results will be achieved in the future with wearable technologies.

## Abbreviations

6MWD: 6-Minute Walk Test distance
6MWT: 6-Minute Walk Test
AUC: area under the curve
FPR: false positive rate
HR: heart rate
HROS: heart rate over steps
HRV: heart rate variability
MVPA: moderate to vigorous physical activity
NET-F: Non-Exercise Testing Cardiorespiratory Fitness
PAL: physical activity level
PPG: photoplethysmography
RHR: resting heart rate
ROC: receiver operating characteristic
TPR: true positive rate
